# Identification of SARS-CoV-2 PLpro and 3CLpro human proteome substrates using substrate phage display coupled with protein network analysis

**DOI:** 10.1016/j.jbc.2023.104831

**Published:** 2023-05-16

**Authors:** Kai Zhao, Yini Li, Mengzhun Guo, Lijia Ma, Bobo Dang

**Affiliations:** 1 College of Life Sciences, Zhejiang University, Hangzhou, Zhejiang, China; 2 Key Laboratory of Structural Biology of Zhejiang Province, School of Life Sciences, Westlake University, Hangzhou, Zhejiang, China; 3 Center for Infectious Disease Research, Westlake Laboratory of Life Sciences and Biomedicine, Hangzhou, Zhejiang, China; 4 Institute of Biology, Westlake Institute for Advanced Study, Hangzhou, Zhejiang, China

**Keywords:** phage display, NGS, substrate specificity, SARS-CoV-2, protease, cadherin

## Abstract

Viral proteases play key roles in viral replication, and they also facilitate immune escape by proteolyzing diverse target proteins. Deep profiling of viral protease substrates in host cells is beneficial for understanding viral pathogenesis and for antiviral drug discovery. Here, we utilized substrate phage display coupled with protein network analysis to identify human proteome substrates of severe acute respiratory syndrome coronavirus 2 (SARS-CoV-2) viral proteases, including papain-like protease (PLpro) and 3C-like protease (3CLpro). We first performed peptide substrates selection of PLpro and 3CLpro, and we then used the top 24 preferred substrate sequences to identify a total of 290 putative protein substrates. Protein network analysis revealed that the top clusters of PLpro and 3CLpro substrate proteins contain ubiquitin-related proteins and cadherin-related proteins, respectively. We verified that cadherin-6 and cadherin-12 are novel substrates of 3CLpro, and CD177 is a novel substrate of PLpro using *in vitro* cleavage assays. We thus demonstrated that substrate phage display coupled with protein network analysis is a simple and high throughput method to identify human proteome substrates of SARS-CoV-2 viral proteases for further understanding of virus–host interactions.

Coronavirus disease 2019 caused by the novel severe acute respiratory syndrome coronavirus 2 (SARS-CoV-2) has been an ongoing pandemic since March 2020 (1, 2). Papain-like protease (PLpro) and 3C-like protease (3CLpro) are key proteases to process viral polyproteins for viral replications (3, 4). Studies in SARS-CoV, Middle East respiratory syndrome–related coronavirus (MERS-CoV), and feline coronavirus (FCoV) revealed that these proteases can cleave host proteins involved in innate immunity and inflammation pathway, facilitating immune escape and viruses spread (5, 6, 7). SARS-CoV-2 PLpro and 3CLpro have also been shown to have similar functions (8, 9, 10).

To better understand the pathogenesis underlying severe pneumonia and the SARS-CoV-2 viral–host interactions, many efforts have been made to identify human protein substrates of these two viral proteases. A systematic screening of 71 human innate immune pathway proteins by *in vitro* protease cleavage assay revealed that PLpro cleaved interferon regulatory factor 3, while 3CLpro cleaved Nod-like receptor pyrin containing domain (NLRP) 12 and TAB1 (9). While the *in vitro* cleavage assay is efficient in identifying putative protein substrates, it is essentially low throughput and cannot identify novel protein substrates beyond the proteins screened.

Affinity purification–based proteomic approaches are powerful and are commonly used to identify interacting proteins; however, they may not be suitable for identifying protease substrates since the affinity between proteases and substrates are generally low. For the same reason, only two putative protein substrates of PLpro and 3CLpro were identified using affinity purification mass spectrometry, further illustrating the limitations of this method (11). In addition, *in silico* methods combining SARS-CoV-2 polyprotein cleavage site analysis with cleavage prediction of putative protein substrates revealed that CTBP1 was cleaved *in vitro* by 3CLpro (12); this method could be significantly improved by replacing limited polyprotein cleavage sequences with much expanded phage library profiled substrate sequences.

Substrate phage display is a powerful method for protease substrates profiling, especially for newly discovered proteases that have been poorly documented (13, 14). Herein, we propose a novel strategy to identify host protein substrates of SARS-CoV-2 viral proteases by coupling the substrate phage display selection with protein network analysis (SPD-PNA). We first constructed a fully randomized heptapeptide phage library to profile the substrate preferences of the SARS-CoV-2 3CLPro and PLpro proteases. We then performed four rounds of substrate phage display selection and used next-generation sequencing (NGS) to identify the substrate sequences. The top 24 sequences were selected for putative human protein substrates identification, followed by protein network analysis in STRING to identify protein clusters. We found that ubiquitin and cadherin (CDH) families were the top substrate protein clusters of PLpro and 3CLpro, respectively. We validated that CDH6 and CDH12 are novel substrates of 3CLpro, and CD177 is a novel substrate of PLpro by *in vitro* cleavage assays.

## Results

### PLpro and 3CLpro substrates selection using phage display

**Figure 1:**
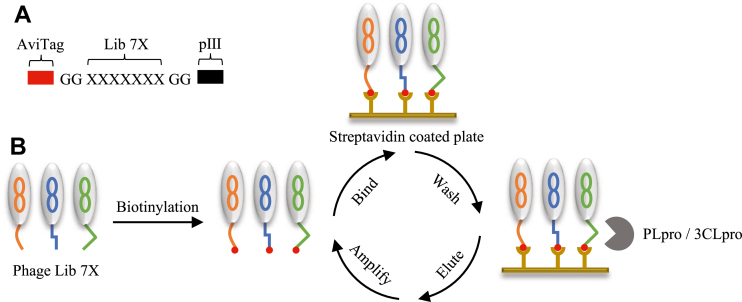
**PLpro and 3CLpro substrates profiling procedures.***A*, the format of the substrate phage library. AviTag: *GLNDIFEAQKIEWHE*. *B*, selection procedure. Phage library was biotinylated through AviTag and then bound to streptavidin-coated plates. After PBST washing, substrate phages were cleaved by PLpro or 3CLpro. The eluted phages were amplified and biotinylated for the next round of selection. 3CLpro, 3C-like protease; PBST, phosphate-buffered saline with Tween 20; PLpro, papain-like protease.

To profile the substrate preferences of SARS-CoV-2 proteases (PLpro and 3CLpro), we initiated the study by constructing a fully randomized heptapeptide library on pIII protein of M13 phage using the phagemid system (Figs. 1*A* and S1) (15). The general procedure of substrate phage display was shown in Figure 1*B*. Before the screening, the input phage (∼10^13^) was labeled with biotin at the N-terminal AviTag for the affinity capture (Fig. S2). The biotinylated phage library was immobilized on the streptavidin-coated plate, and unbound phages were washed away with phosphate-buffered saline with Tween 20 buffer. The bound phages were incubated with PLpro or 3CLpro in HEPES buffer for corresponding substrate phage release. Released phages were then amplified for the next round of selection. To enrich the preferred substrates, we shortened the protease incubation time from 3 h to 15 min over four rounds. We also performed a blank selection where protease cleavage elution was replaced with HEPES buffer washing.

**Figure 2:**
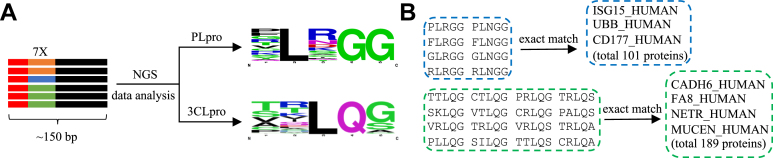
**Profiled substrate sequences analysis and putative human protein substrates of PLpro and 3CLpro.***A*, barcodes (*red*) were inserted at the terminal of the heptapeptide gene (*orange*, *blue*, and *green*) by PCR with barcoded forward primers for NGS analysis. Based on the NGS data, the extracted substrate sequences of PLpro and 3CLpro were analyzed using WebLogo. *B*, top sequences from seqlogo of PLpro (eight sequences) and 3CLpro (16 sequences) were selected to identify putative protein substrates in UniProt’s human proteome. 3CLpro, 3C-like protease; NGS, next-generation sequencing; PLpro, papain-like protease.

After the selection, we sequenced the selected phages by NGS (0.1 ∼ 0.15 million reads for each sample, Table S1). We then ranked all the sequences based on the enrichment score (enriched sequence read count in the PLpro/3CLpro group divided by the read count of the same sequence in the Hepes buffer control group). The enriched peptide sequences were extracted by signature sequence (for PLpro, enrichment score ≥ 50, “XLXGG”; for 3CLpro, enrichment score ≥ 100, “XXLQX”) for seqLogo analysis (Fig. 2*A*). To find protein substrates of SARS-CoV-2 proteases in the human proteome, we picked putative protein substrates containing the selected top 8 substrate sequences of PLpro and top 16 substrate sequences of 3CLpro in UniProt’s human proteome database (UP000005640). We found a total of 101 and 189 human proteins as putative substrates of PLpro and 3CLpro, respectively (Fig. 2*B* and Table S2).

### Protein network analysis of putative protein substrates

**Figure 3:**
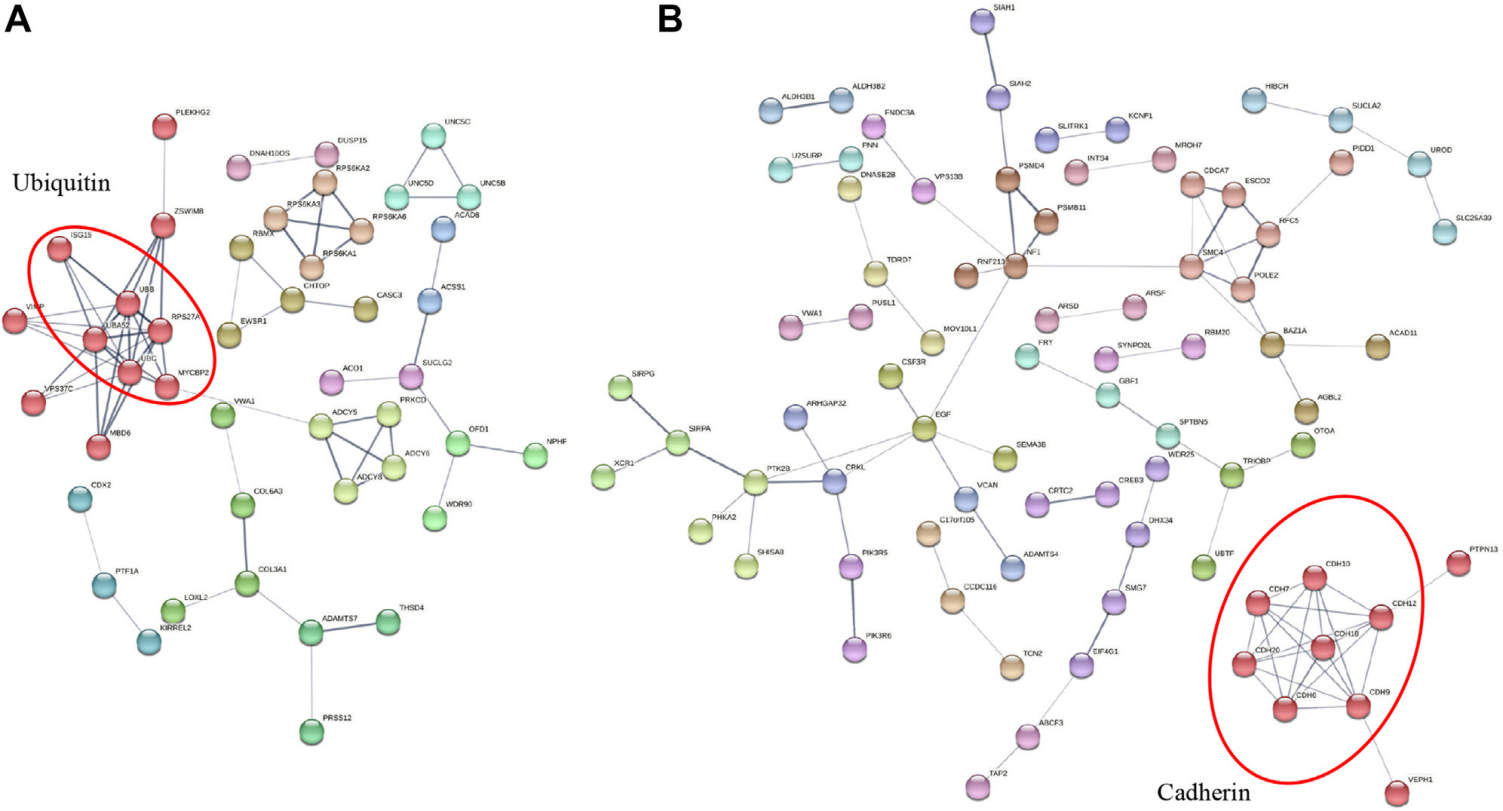
**Protein network analysis of PLpro and 3CLpro putative protein substrates.** *A*, Protein network analysis of PLpro putative protein substrates*. B*, Protein network analysis of 3CLpro putative protein substrates. Protein network analysis was performed by STRING analysis and Markov Clustering. Disconnected nodes were hidden in the network. Line thickness indicated the strength of confidence. After clustering, the top cluster (*red balls*) was circled. 3CLpro, 3C-like protease; PLpro, papain-like protease.

Protein network analysis can show the classification of protein families and reveal the global connection between the proteins based on their protein–protein interactions. The names of the 101 putative protein substrates (Table S1) of PLpro were further uploaded into STRING for network analysis, followed by Markov Clustering. As shown in Figure 3*A*, the top cluster contains six ubiquitin-related proteins (ISG15, RPS27A, UBA52, polyubiquitin-B [UBB], polyubiquitin-C [UBC], and MYCBP2). Ubiquitin-like protein ISG15 (ISG15), UBB, and UBC have been validated as substrates of PLpro (8). As for 3CLpro, 189 putative protein substrates (Table S1) were analyzed by STRING with the same procedure. Seven core proteins (CDH10, CDH12, CDH18, CDH20, CDH6, CDH7, and CDH9) in the top cluster belong to the CDH family (Fig. 3*B*). CDH6, CDH20, and RNF213 have recently been reported to be the substrates of 3CLpro (16, 17), which corroborates our finding here. These results together demonstrate that SPD-PNA strategy is efficient in identifying human proteome substrates of SARS-CoV-2 proteases.

### Validation of protein substrates

**Figure 4:**
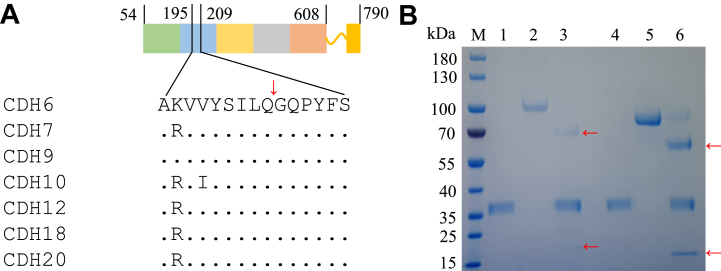
**3CLpro cleavage of CDH6 and CDH12.***A*, sequence alignment of selected cadherin proteins; CDH6 contains five cadherin domains (54–608), “.” indicates the same amino acid compared with CDH6, “↓” indicates the cleavage site. *B*, CDH6 and CDH12 cleavage analysis. Lane M: protein marker, lane 1: 3CLpro; lane 2: CDH6; lane 3: 3CLpro + CDH6, incubation at 37 °C for 4 h, “←” indicates the cleaved CDH6; lane 4: 3CLpro; lane 5: CDH12; lane 6: 3CLpro + CDH12, incubation at 37 °C for 4 h, “←” indicates the cleaved CDH12. 3CLpro, 3C-like protease.

**Figure 5:**
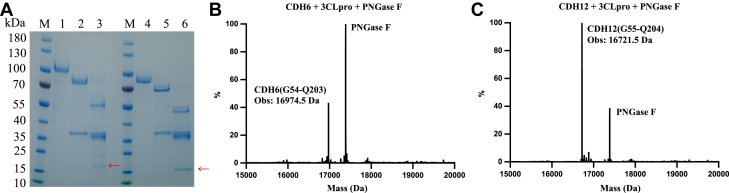
**CDH6 and CDH12 cleavage sites confirmation.***A*, lane M: protein marker, lane 1: CDH6, lane 2: CDH6 + PNGase F, lane 3: CDH6 + PNGase F + 3CLpro, lane 4: CDH12, lane 5: CDH12 + PNGase F, lane 6: CDH12 + PNGase F + 3CLpro, “←” indicates the cleaved N-terminal fragments of CDH6 and CDH12. *B* and *C*, mass spectrometry analysis; the calculated mass of CDH6 (G54-Q203) is 16974.8 Da, and the calculated mass of CDH12 (G55-Q204) is 16721.6 Da. 3CLpro, 3C-like protease; CDH6, cadherin-6; PNGase F, peptide PNGase F.

Multiple sequence alignment analysis of all seven cadherin proteins showed high sequence similarity and the same predicted cleavage site sequence “SILQG” (Fig. 4*A*). We chose the commercially available CDH6 and CDH12 to test whether they are indeed the substrates of 3CLpro. We coincubated CDH6 or CDH12 with 3CLpro for 4 h at 37 °C and found these two proteins can indeed be cleaved by 3CLpro (Fig. 4*B*). To further confirm the cleavage site, we used mass spectrometry to analyze the exact mass of the cleaved and deglycosylated N-terminal fragment of CDH6 and CDH12 (Fig. 5). The observed mass matched exactly with the calculated mass of the cleaved N-terminal fragment of CDH6 and CDH12, which demonstrated that the cleavage site is consistent with the prediction (SILQ↓G).

**Figure 6:**
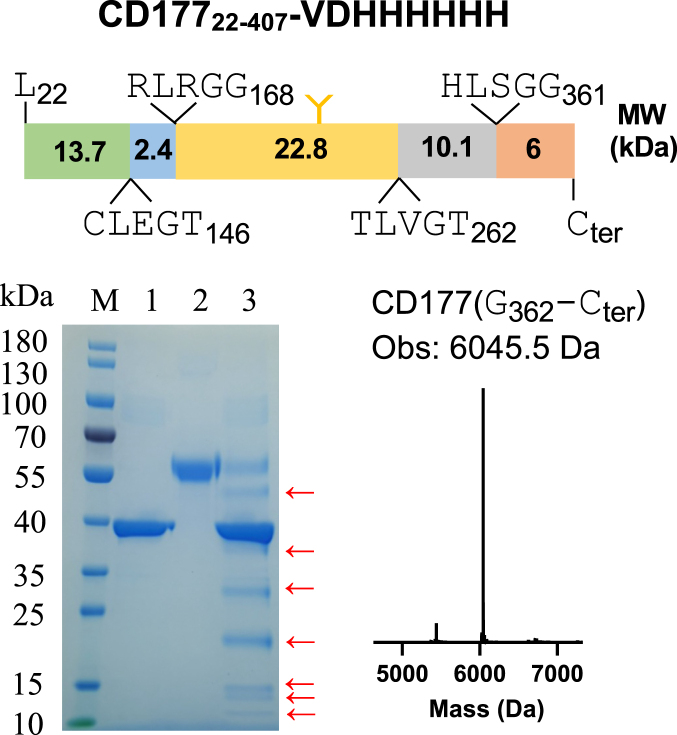
**PLpro cleavage of CD177.** Multiple predicted cleavage sites are shown in the CD177 diagram based on SDS-PAGE analysis. Lane M: protein marker, lane 1: PLpro; lane 2: CD177; lane 3: PLpro + CD177, incubation at 30 °C for 12 h, “←” indicates the cleaved CD177. Mass spectrometry data shown here corresponds to one of the cleaved CD177 fragments (G_362_-C_ter_, Calc. 6045.7 Da, Obsd. 6045.5 Da). PLpro, papain-like protease.

Given that ISG15 from the ubiquitin family has been identified as a substrate of PLpro (8), we sought to identify other potential substrates for further investigation. Upon examining different substrate protein structures, we found CD177 predicated cleavage site locates in a flexible loop region; we thus selected CD177 for *in vitro* cleavage validation. Twelve hours of coincubation of CD177 with PLpro at 30 °C revealed that CD177 was cleaved by PLpro at multiple sites (Fig. 6). One of the cleavage sites (HLSGG↓) was confirmed by the exact mass of fragment G_362_-C_ter_.

## Discussion

To simplify our data analysis, we only used the top-ranked substrate sequences for protein network analysis, and we were not able to uncover all previously validated protein substrates within this analysis (8, 9, 10, 18, 19). We wonder if all these previously validated protein cleavage site sequences can be found within the whole list of our profiled substrate sequences. After searching, we indeed found all these sequences with distinct enrichment scores (Table 1). We then synthesized some of these substrate peptides with distinct enrichment scores and demonstrated that 3CLpro preferably cleaved substrate sequences with higher enrichment scores (Fig. S3). Since we only selected top sequences with high enrichment scores, several previously known substrates were not identified in our initial analysis. We then performed a protein network analysis of our profiled protein substrates with these previously validated protein substrates included (Fig. S4). The results revealed that NLRP1 found in our study and previously validated NLRP6, and NLRP12 belong to the Nod-like receptor family, which interact with other previously validated GSDMD, IKBKG, and TAB1 proteins. We also found previously validated NOTCH1 and F2 interact with the EGF protein found in our study. In addition, previously validated interferon regulatory factor 3 was also clustered within the ubiquitin-related proteins in our analysis. These results collectively demonstrate SPD-PNA can efficiently identify novel PLpro and 3CLpro protein substrates.

**Table 1:** Validated human protein substrates of PLpro and 3CLpro

SARS-CoV-2 protease	Substrates in human proteome	Cleavage site	Enrichment score^a^ in the fourth round
3CLpro	CDH6	SILQG	464
	CDH12	SILQG	464
	NLRP1	VILQG	268
	NOTCH1	SRLQS	164
	GSDMD	TCLQG	40
	NLRP12	VVLQA	19
	TAB1	LTLQS/ASLQS	15/7
	F2	ASLQA	6
	IKBKG	AQLQV	2
PLpro	CD177	HLSGG	50
	UBB	RLRGG	29
	ISG15	RLRGG	29
	IRF3	CLGGG	19

a Enrichment score = (reads of sample + 1)/(reads of control + 1).

Viral proteases are essential for the replication and propagation of viruses, they also employ proteolytic activity to interact with host cells to disrupt immune responses, thus facilitating viruses spread. Given many human proteins potentially can be substrates of viral proteases, it is difficult to profile these human proteins in depth using traditional synthetic peptide libraries or *in silico* analysis based on limited sequence libraries. Substrate phage display is advantageous in deep profiling protease substrates since it can display a much larger peptide library and the selected substrate sequences can be analyzed using NGS, thus allowing complete and sensitive substrates profiling (20, 21). Herein, we employed substrate phage display and NGS to identify the peptide substrates of SARS-CoV-2 PLpro and 3CLpro. We used the top 24 substrate sequences to identify putative protein substrates in the human proteome. Limited by the available resources, we only applied protein network analysis to the key protein substrates for validation. Notably, we found the previously validated PLpro substrates, including ISG15, UBB, and UBC in our protein network analysis. We also validated CDH6 and CDH12 are new substrates of 3CLpro, and CD177 is a novel substrate of PLpro. The fact that many previously reported protein substrates could be found in our analysis demonstrates the power and efficiency of this method. We believe the SPD-PNA strategy is a valuable method and can efficiently complement other approaches including affinity purification mass spectrometry proteomic for identifying proteome substrates of proteases.

## Experimental procedures

### Construction of substrate phage library

Lib 7X was constructed by Kunkel mutagenesis (22). Firstly, dU-ssDNA was harvested from uridine medium of *Escherichia coli* CJ236 harboring the phagemid template. After the annealing of the phosphorylated primers to dU-ssDNA, a heteroduplex CCC-dsDNA was synthesized by fill-in reaction with T7 DNA polymerase and T4 DNA ligase. Then the CCC-dsDNA was electroporated into electrocompetent TG1 (Lucigen) to produce the phage library.

### Substrate phage selection of PLpro and 3CLpro

To label biotin on M13 phages, BirA enzyme (0.06 U/μl) was used to ligate biotin on AviTag with 3 mM ATP in biotinylation buffer (50 mM Tris, 5 mM MgCl_2_, and 1 mM biotin, pH 8.0) at 4 °C overnight. Then the biotinylated phages (∼1 × 10^13^) were loaded on streptavidin-coated plate with gently shaking for 2 h at 25 °C. After washing with phosphate-buffered saline with Tween 20 for 12 times, 50 nM 3CLpro or PLpro in 20 mM Hepes buffer was added at 37 °C to elute substrate phages for varied times (3 h, 2 h, 0.5 h, and 15 min). The cleaved phages were recovered to infect TG1 (*A*_600_ ∼ 0.5) and plated on 2YT/Amp plates (150 mm diameter) to culture at 37 °C overnight. TG1 cells were then recovered to amplify phages for the next round panning. A blank selection with Hepes buffer washing instead of protease cleavage elution was also performed to facilitate enrichment score analysis after NGS.

### NGS sample preparation and data analysis

With extracted DNA from each round of selection in hand, the fragments coding 7X peptide were amplified with primers containing barcode. The PCR products (∼150 bp) were ligated with dual adapters as a mixed library for NGS by Illumina 150PE (GENEWIZ).

The sequencing data were processed with custom script to extract 21 bp DNA sequences coding heptapeptide based on the inserted barcode in each sample. Then the DNA sequences were translated into protein sequences according to human codon table. We calculated the enrichment score for each protein sequence with offset 1 ([reads of sample + 1]/[reads of control + 1]). After that, we ranked the protein sequences based on the enrichment score. The profiled substrate preferences of 3CLpro and PLpro were further analyzed by WebLogo.

To identify PLpro and 3CLpro human proteome substrates, we downloaded the human proteome database from UniProt website (https://www.uniprot.org/proteomes/UP000005640) and picked putative protein substrates which contain the selected top 24 substrate sequences. With putative protein substrates in hand, we further performed protein network analysis by STRING. In detail, we uploaded names of all the putative proteins and searched for protein-protein interaction network. To simplify the mapping, we hid disconnected nodes in the network and chose line thickness to indicate the strength of confidence. Then we further performed Markov Clustering to classify the protein families with inflation parameter 3.

### Protein substrates verification assay *in vitro*

CDH6 (Sino Biological, 10150-H08H), CDH12 (Sino Biological, 10317-H08H), and CD177 (Novoprotein, C442) *in vitro* cleavage assay was performed in house. In detail, 2 μM 3CLpro was added to cleave 2 μg CDH6 or CDH12 in 50 mM PBS buffer (pH 7.4) at 37 °C for 4 h. Five micromolar PLpro was added to cleave 3 μg CD177 in 20 mM Hepes buffer (pH 7.4, 150 mM NaCl, 1 mM DTT, and 0.5 mM EDTA) at 30 °C for 12 h. Then the cleavage was terminated by boiling with protein loading buffer, followed by SDS-PAGE analysis.

### Cleavage site confirmation assay

The cleavage site of CDH6 and CDH12 could be confirmed by exact mass of the cleaved N-terminal fragment. Peptide PNGase F was used to remove the N-linked glycosylation; we incubated 3 μg CDH6 or CDH12 with 1 μl peptide PNGase F (NEB, P0704S) and 5 μM 3CLpro in 50 mM sodium phosphate (pH 7.5) at 37 °C for 12 h. After incubation, 0.1 μg of CDH6 or CDH12 was injected into high resolution mass spectrometer (Waters/SYNAPT XS HDMS) for analyzing the cleaved N-terminal fragment. For CD177, we simply injected the reaction solution of the CD177 cleavage assay for mass spectrometry analysis.

## Data availability

All experimental data for this article are available upon email request to: Bobo Dang (dangbobo@westlake.edu.cn).

## Supporting information

This article contains supporting information.

## Conflict of interest

The authors declare that they have no conflicts of interest with the contents of this article.
