# Supplementary material for: Identification of SARS-CoV-2 PLpro and 3CLpro human proteome substrates using substrate phage display coupled with protein network analysis
Source: J Biol Chem. 2023 May 16;299(6):104831. doi: 10.1016/j.jbc.2023.104831 (PMC10185492; doi:10.1016/j.jbc.2023.104831)
Supplement: Figures S1–S4 [file mmc2.docx]

**Supporting information for**

**Identification of SARS-CoV-2 PLpro and 3CLpro human proteome substrates using substrate phage display coupled with protein network analysis**

*Kai zhao^1,2,3,4^, Yini Li^2,3,4^, Mengzhun Guo^2,3,4^, Lijia Ma^2,3,4^, Bobo Dang^1,2,3,4^**

1. College of Life Sciences, Zhejiang University, Hangzhou, Zhejiang, China

2. Key Laboratory of Structural Biology of Zhejiang Province, School of Life Sciences, Westlake University, Hangzhou, Zhejiang, China

3. Center for Infectious Disease Research, Westlake Laboratory of Life Sciences and Biomedicine, Hangzhou, Zhejiang, China

4. Institute of Biology, Westlake Institute for Advanced Study, Hangzhou, Zhejiang, China

* For correspondence: Bobo Dang, [dangbobo@westlake.edu.cn](mailto:bobodang@westlake.edu.cn).

**Contents:**

Figure S1-S4

Table S1-S2


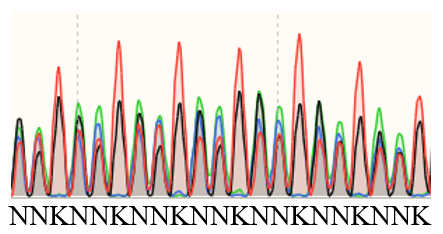


**Figure S1. Sanger sequencing of initial Lib 7X.** N = A, C, G, T; K = G, T.

**Figure S2. Verification of biotinylated phage library.** ***A***, Lane M: Marker, Lane 1, 2 initial biotinylated naïve phage Lib 7X (these two lanes have the same sample); ***B***, Lane M: Marker, lane 1: biotinylated phages in 3CLpro group after 1^st^ round selection, Lane 2: biotinylated phages in PLpro group after 1^st^ round selection, Lane 3: biotinylated phages in HEPES group after 1^st^ round selection; ***C***, Lane M: Marker, lane 1: biotinylated phages in 3CLpro group after 2^nd^ round selection, Lane 2: biotinylated phages in PLpro group after 2^nd^ round selection, Lane 3: biotinylated phages in HEPES group after 2^nd^ round selection; ***D***, Lane M: Marker, lane 1: biotinylated phages in 3CLpro group after 3^rd^ round selection, Lane 2: biotinylated phages in PLpro group after 3^rd^ round selection, Lane 3: biotinylated phages in HEPES group after 3^rd^ round selection.


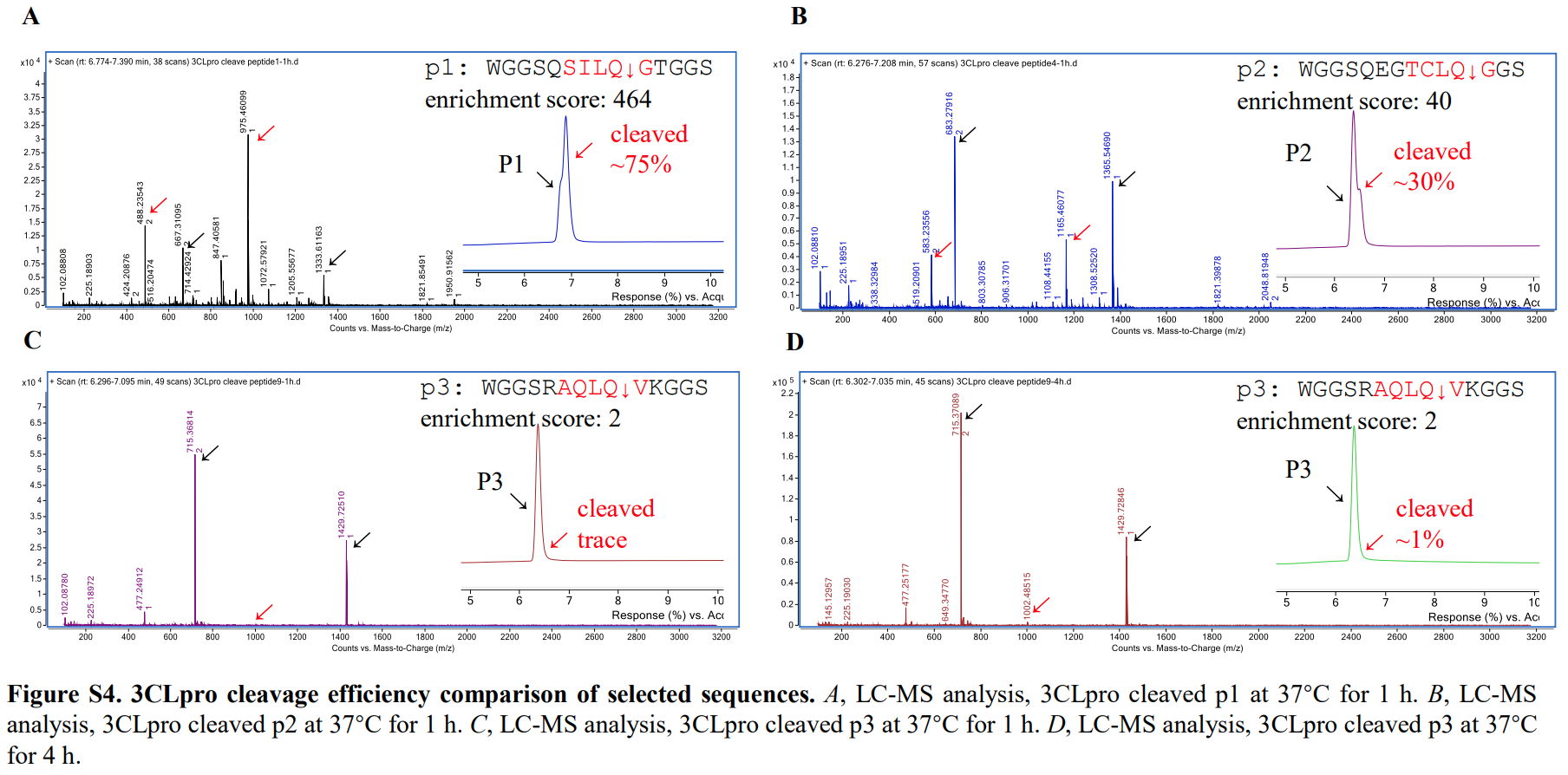


**Figure S3.** 3CLpro cleavage efficiency comparison of selected sequences. ***A***, LC-MS analysis, 3CLpro cleaved p1 at 37°C for 1 h. ***B***, LC-MS analysis, 3CLpro cleaved p2 at 37°C for 1 h. ***C***, LC-MS analysis, 3CLpro cleaved p3 at 37°C for 1 h. ***D***, LC-MS analysis, 3CLpro cleaved p3 at 37°C for 4 h. Black arrow indicates uncleaved peptides, red arrow indicates cleaved peptides.

**Figure S4. Protein network analysis using profiled and validated protein substrates of PLpro (A) and 3CLpro (B).** Protein network analysis was performed by STRING analysis with names of multiple proteins and Markov Clustering. Disconnected nodes were hidden in the network. Line thickness indicated the strength of confidence. The validated protein substrates are pointed out with red arrows.
